# Refractory Dysmenorrhea Managed With a Glucagon-Like Peptide-1 Agonist: A Case Report

**DOI:** 10.7759/cureus.77387

**Published:** 2025-01-13

**Authors:** Mary Tran, Nicholas Swartz, Sabine D Elisèe

**Affiliations:** 1 Internal Medicine, Nova Southeastern University Dr. Kiran C. Patel College of Osteopathic Medicine, Davie, USA; 2 Family Medicine, Cornerstone Medical Group, Coral Springs, USA

**Keywords:** glucagon-like peptide 1, menstrual pain, pain in women, primary dysmenorrhea, semaglutide efficacy, severe dysmenorrhea, women’s health

## Abstract

Dysmenorrhea includes symptoms such as abdominal pain, nausea, and vomiting. It is a clinical diagnosis with typical treatment involving the use of non-steroidal anti-inflammatory drugs (NSAIDs) and hormonal therapy. Even with conventional treatment, many women deal with dysmenorrhea refractory to treatment. They are often subject to dealing with the adverse effects of those treatments, such as peptic ulcer disease. We present a case of a patient with severe refractory dysmenorrhea who was able to minimize symptoms with the use of semaglutide, a glucagon-like peptide-1 (GLP-1) agonist. With no changes in the patient’s lifestyle and no additional medications added, the semaglutide therapy was able to minimize the patient’s symptoms to where she was able to continue activities of daily living and minimize the use of NSAID therapy. Moreover, the patient experienced no changes in weight and only transient loss of appetite while on semaglutide. We hypothesize that anti-estrogenic and anti-inflammatory properties are potential mechanisms of action in this case. This case brings to light the possible multifaceted applications of GLP-1 agonists beyond weight control and diabetes. The outcome of this case suggests that a controlled trial of GLP-1 agonist therapy is warranted to determine its potential for the management of dysmenorrhea.

## Introduction

The use of glucagon-like peptide-1 (GLP-1) agonists, such as semaglutide and tirzepatide, has recently gained significant public attention for their efficacy in treating obesity and promoting weight loss [1,2]. These medications act as incretin hormone mimetics that maintain glucose homeostasis by increasing insulin secretion, reducing food intake, decreasing glucagon secretion, and reducing gastric emptying [3]. As a treatment for type 2 diabetes and obesity, semaglutide can be implicated as a treatment for female reproductive syndromes surrounding insulin resistance and obesity. Although originally developed for the management of type 2 diabetes, several off-label uses are being explored for this class of medications. One potential off-label application is the treatment of dysmenorrhea, which affects almost 50% of reproductive-aged women [4]. 

Dysmenorrhea can be associated with severe pelvic cramps and even nausea and vomiting as seen in this case [4]. Refractory dysmenorrhea is menstrual pain unresponsive to treatment, with about 18% of women with dysmenorrhea being unresponsive to non-steroidal anti-inflammatory drugs (NSAIDs) [4]. Dysmenorrhea is thought to be a result of prostaglandins released during the secretory phase of the menstrual cycle [5]. These prostaglandins are produced and released by the endometrial lining [6]. There is a positive correlation between the quantity of serum prostaglandins and the severity of the associated dysmenorrhea symptomatology [5]. Notably, adipose tissue converts androgens to estrogen, leading to an increased estrogenic state in those with obesity [6]. This increased estrogenic state is believed to play a role in the increased thickness of the endometrial lining, thus the increased levels of prostaglandins released during the secretory phase of menstruation [6].

Current medical therapies for dysmenorrhea primarily focus on symptomatic management with other over-the-counter analgesics and hormonal modulation [7,8]. For many, these therapies do not provide the relief they are seeking or pose as nonviable options due to various personal reasons. Alternative treatment options are highly desirable for both patients and healthcare providers; GLP-1 agonists possibly being one such alternative for the treatment of dysmenorrhea [9]. We present a case in which severe dysmenorrhea refractory to NSAID therapy was successfully managed with 0.25 mg subcutaneous semaglutide every two weeks. This decision was based on a previous patient of the treating physician who had improvement in menstrual symptoms while on this medication for physician-guided weight loss. This unintended improvement in pain associated with menses prompted the treating physician to try it as therapy for the dysmenorrhea of this case’s patient.

## Case presentation

In December 2023, a 20-year-old female patient, gravida 0 para 0, with no pertinent medical, surgical, or family history, presented to the outpatient family medicine office with a complaint of severe dysmenorrhea. She reported having environmental allergies to pollen and dust; otherwise, she denied any allergies. She reported experiencing pelvic pain, vomiting, nausea, and syncope associated with menses. The patient reported experiencing significant dysmenorrhea since the onset of menses at the age of 11 years. These symptoms would last three to four days. She rated the severity of her symptoms as 12/10 and noted that she was having a hard time completing activities of daily living due to the symptoms. Her cycles were 29-32 days long, and her menses lasted five to seven days. She reported heavy bleeding for most days with the last one to two days of spotting or light bleeding. She reported her last menstrual period occurred two weeks before her office visit. The patient had seen four different obstetrics and gynecology specialists about her symptoms in the past and was directed to use a warm compress and NSAID therapy. She denied ever being diagnosed with polycystic ovarian syndrome (PCOS) or any other gynecological disorders. She also denied completing any imaging or lab work. The patient was taking 200 milligrams of ibuprofen three times a day for two to three days of her cycle. She reported that ibuprofen helped minimally with her symptoms. She reported also trying acetaminophen and an acetaminophen/pamabrom/pyrilamine combination pill for pain relief, which minimally helped with her symptoms. 

Physical examination revealed stable vital signs (Table 1). Of note, her BMI was 21.1 kg/m², with a healthy BMI range being 18.5-24.5 kg/m². The abdominal exam showed positive normoactive bowel sounds, nontender to palpation, no masses, and no hepatosplenomegaly. Lungs were clear to auscultation bilaterally with no accessory breathing. The heart exam revealed a 3/6 asymptomatic systolic murmur; otherwise, the heart exam showed a regular rate and rhythm. Other pertinent physical exam findings included dry, warm skin and a supple, non-tender thyroid. A pelvic exam was not conducted. Based on clinical presentation and history of present illness, the patient was diagnosed with dysmenorrhea. This diagnosis was deemed fit based on the clinical presentation of the patient and with the support of no abnormal physical exam findings. The use of compounded semaglutide for the control of her menstrual symptoms was discussed, and the decision to treat with semaglutide was made by the treating physician based on the outcome of one previous patient.

**Table 1:** The patient's vital signs taken during her two in-office visits Vitals to note: the patient’s oxygen saturation was 89% at her initial in-office visit but denied any symptoms at this time. Her blood pressure and heart rate both decreased at the subsequent visit. The patient did not have any complaints following this change in her vitals, including any headache or dizziness. These changes could be attributed to errors in collecting and documenting vitals.

	Visit in December 2023	Visit in January 2024
Blood pressure (mmHg)	118/62	82/52
Heart rate (beats per minute)	85	58
Temperature (degrees Fahrenheit)	97.7	97.8
Weight (pounds)	135	135
Height (inches)	67	67
BMI (kg/m^2^)	21.1	21.1
Oxygen saturation	89	97

In January 2024, the patient was seen and instructed on how to start subcutaneous self-injections of the compounded semaglutide. She was instructed to self-administer 0.25 mg on the third and 17^th^ of each month. On this office visit day, she received her first dose of 0.25 mg semaglutide, and she was started on vitamin D 50,000 units once weekly for general health. The patient returned for a follow-up in early February of 2024 via video call and reported a significant decrease in her menstrual symptoms of pelvic pain, vomiting, nausea, and syncope. She noted only experiencing mild pelvic cramping. She rated the severity as 3/10. She noted that she was able to travel and participate in her activities of daily living. She reported only having to take two ibuprofen total during her last menstrual period, which occurred in mid-January of 2024. Since starting the treatment, she denied any changes in her diet or exercise routine. The patient initially experienced decreased appetite with the start of the compounded semaglutide but returned to baseline. She started having daily bowel movements and did not report any other adverse effects. She denied any changes to her weight from her weight taken at her last in-office visit (Table 1).

During the late spring of 2024, she stopped the compounded semaglutide for about three months due to running out of the medication. During this time, she reported that all of her menstrual-associated symptoms returned, including pelvic pain, nausea, vomiting, and syncope. The patient noted that the severity progressively worsened with each month off of the medication. Furthermore, she stated the severity returned to baseline as it was before she started the semaglutide. She reported experiencing an extreme increase in appetite and weight gain. After being off of the compounded semaglutide for three months, the patient resumed treatment at 0.25 mg twice a month as directed. She reported that her menstrual symptoms regressed to minimal cramping with subsequent menses after she resumed treatment. She also was able to return to her baseline weight.

In early October 2024, the patient was contacted via phone call for a status update. She continued to take compounded semaglutide 0.25 mg twice a month as directed. She was no longer taking vitamin D but was taking an oral probiotic. She reported that her menstrual symptoms were stable and reported only experiencing mild pelvic cramping. She reported rarely needing ibuprofen for the pain and noted that she had not taken any pain medication for the past two menstrual cycles. The patient reported that her length of menses had decreased from an average of seven days to four days. She continued with her usual diet and exercise with no changes. She denied any nausea, vomiting, syncope, appetite changes, or weight changes.

## Discussion

Refractory dysmenorrhea, characterized by pelvic pain during menstruation that is unresponsive to conventional treatments, can be challenging to manage. As seen in this case, the standard treatment with NSAIDs failed, leading to the patient being unable to complete her activities of daily living, which diminished her quality of life. The patient was experiencing such severe symptoms that she had to take an average of nine pills of 200 milligrams of ibuprofen in a three-day time period, only for minimal relief. Hormonal therapies, another conventional treatment, were not a viable option for this patient due to personal reasons, leaving her with few options. GLP-1 agonists have been explored as a therapeutic agent for PCOS, namely due to their metabolic and fertility benefits in individuals with PCOS [10,11]. In the same way, GLP-1 agonists can be implicated in the treatment of dysmenorrhea due to their anti-obesity and thus indirect anti-estrogenic action [3,12]. With the use of compounded semaglutide, the patient in this case’s menstrual symptoms decreased and led to minimal use of NSAID therapy. 

As aforementioned, increased adipose tissue is associated with an increased estrogenic state. Increased estrogen plays a role in increased thickness of the endometrial lining and higher levels of prostaglandins, the inflammatory marker responsible for pain, released during the secretory phase of menstruation [13,6]. This proposes that decreasing weight and reducing estrogenic state could be a mechanism by which the compounded semaglutide helps decrease dysmenorrhea symptoms. However, the patient described in this case did not lose weight and has not noticed any overall weight loss since starting the semaglutide treatment. Despite not losing any weight, the patient maintains a markedly decreased severity in her dysmenorrheic symptoms since starting treatment. To note, the patient was not taking any other medications besides vitamin D and probiotic supplements. She also did not change anything in her diet or exercise routine. This eliminates any confounding factors regarding lifestyle factors. Recall that with previous gynecology specialist visits, she did not receive a diagnosis of PCOS or any other gynecological disorder. It is essential to note the importance of ruling out such gynecological disorders in cases of dysmenorrhea since the pain associated with menstruation can be secondary to other gynecological conditions.

This leads to considering anti-inflammatory properties as the mechanism by which GLP-1 agonists can help manage dysmenorrhea. GLP-1 agonists have a role in controlling immune cell signaling [14]. This includes cells such as macrophages, monocytes, and lymphocytes, all of which are involved in the release of inflammatory cytokines [14]. In those with type 2 diabetes, it was observed that those treated with a GLP-1 agonist had decreased levels of prostaglandin and cytokine interleukin-6 levels [14]. It was also observed that prostaglandins induced interleukin-6 production itself, indicating that prostaglandins are an important mediator of inflammation [15]. This shows that GLP-1 agonists act as direct anti-inflammatory modulators. The anti-inflammatory property of GLP-1 agonists shows its potential indications for cases where the mechanism of disease is inflammation, as seen in dysmenorrhea [14]. This could be the potential mechanism by which the GLP-1 was able to help minimize this patient’s symptoms, considering she noticed the benefits with just two doses of the medication and experienced no weight changes. 

After starting the GLP-1 agonist, the patient unintentionally stopped taking the medication for about three months, during which she noticed that her symptoms returned to the same severity as before starting treatment. During this time she also noticed an increase in appetite and weight, this being a common side effect of stopping GLP-1 agonist treatment [16]. When the patient restarted treatment, her symptoms drastically decreased to a minimum and continued to be under control thereafter. The patient noted that her appetite and weight also went back to baseline. This unintentional trial of the medication and her not changing anything in her routine can serve as evidence that her outcomes can be attributed to the GLP-1 agonist therapy. Additionally, she reports no side effects with the 0.25 mg dose twice-monthly regimen, other than the initial transient loss of appetite. A twice-a-month dosing frequency, rather than the conventional once-a-week dosing commonly used for diabetes or weight loss, was chosen because the goal was to manage her dysmenorrhea, not to promote weight loss. Furthermore, starting the patient on the lowest dosage at a twice-a-month frequency was done to help minimize the risk of adverse reactions and allowed for gradual adjustments if necessary. 

It is important to note that pancreatitis, medullary thyroid cancer, and multiple endocrine neoplasia syndrome type 2 (MEN2) are several contraindications for GLP-1 agonist use [17]. GLP-1 agonists can also cause adverse effects such as gastroparesis and bowel obstruction, with a case showing symptoms starting at 0.5 mg once-weekly dosage and frequency [17,18]. These contraindications limit which patients this therapy can be a viable option for. Comparatively, chronic NSAID use has potential adverse effects, including peptic ulcer disease and renal failure [19,20]. Again, the potential adverse effects of NSAID therapy can limit which patients it can benefit. Additionally, as seen in this case, NSAID therapy may not be effective enough to manage symptoms. Once more, this underlines GLP-1 agonist as an alternative option for treating dysmenorrhea in cases where conventional treatment is ineffective or is not an option. 

Considering GLP-1 agonists’ observed potential mechanisms of action as indirect anti-estrogenic and direct anti-inflammatory therapy, it is possible to consider them for future use as an alternative treatment in cases of severe or refractory dysmenorrhea. A randomized clinical trial is necessary to confirm these findings and determine whether GLP-1 agonist therapy is appropriate in patients with recurrent or refractory dysmenorrhea.

## Conclusions

In summary, this is a case of severe dysmenorrhea refractory to NSAID use that was successfully managed using GLP-1 agonist therapy. The patient experienced a substantial decrease in her symptoms and was able to continue with her activities of daily living. The literature suggests that the possible mechanisms by which the GLP-1 agonist was able to manage the patient’s symptoms are its anti-inflammatory and anti-estrogenic effects. The significant improvement in this patient's menstrual symptoms suggests that GLP-1 agonist therapy can serve as an alternative therapy where conventional treatments are not optimal. This case highlights GLP-1 agonists as a potential mainstream therapy for dysmenorrhea and shows that more research is needed to confirm these findings.
